# CEUS in Atypical Renal Cystic Masses: How, When and Why

**DOI:** 10.3390/medicina62040721

**Published:** 2026-04-09

**Authors:** Michele Bertolotto, Irene Campo, Alessandra Oliva, Antonio Granata, Vito Cantisani

**Affiliations:** 1 Unit of Training & Research in Vascular & Multiparametric US, Clinical Department for Medical, Surgical and Health Sciences—University of Trieste, Cattinara Hospital, Strada di Fiume 447, 34149 Trieste, Italy; alessandra_oliva@icloud.com; 2 Department of Radiology, University of Trieste, San Giovanni di Dio Hospital, Via Fatebenefratelli 34, 34170 Gorizia, Italy; irenecampo11@gmail.com; 3 Division Nephrology and Dialysis Unit, Cannizzaro Hospital, Via Messina 829, 95126 Catania, Italy; antonio.granata4@tin.it; 4 Internal Medicine and Surgical Ultrasound Unit, Department of Radiological, Oncological, and Anatomopathological Sciences, Umberto I Hospital, Sapienza University, 00161 Rome, Italy; vito.cantisani@uniroma1.it

**Keywords:** renal cyst-ultrasound, renal cyst-Bosniak classification, renal cyst-contrast-enhanced ultrasound

## Abstract

*Background/Objectives*: Cysts are the most common kidney lesions identified in patients undergoing abdominal imaging, with ultrasound (US) typically serving as the initial diagnostic tool. Contrast-enhanced ultrasound (CEUS) has emerged as a highly effective modality for the evaluation of cystic renal lesions, particularly when conventional B-mode ultrasound (US) or CE-CT are inconclusive. While simple renal cysts are readily characterised on US, cystic renal lesions require further assessment. *Methods*: The Bosniak classification, originally developed for CE-CT, remains the cornerstone for categorising cystic renal lesions, guiding management from surveillance to surgical intervention. Recent efforts to standardise CEUS-specific imaging parameters and adapt the Bosniak criteria aim to improve interobserver agreement, reduce subjectivity, and enhance diagnostic accuracy. *Results*: CEUS offers superior sensitivity for detecting slow blood flow and minimal vascularity within septa, wall or solid components, often outperforming CE-CT in real-time vascular assessment. However, the high sensitivity of CEUS can reveal additional septa or subtle enhancement, potentially leading to lesion overscoring, if the different sensitivity of CEUS and CT/MRI for detecting enhancement is not taken into account. CEUS also plays a crucial role in the follow-up of non-surgical cystic lesions, providing a radiation-free and cost-effective alternative for long-term monitoring. Certain scenarios, such as post-interventional changes, traumatic cystic rupture, or infected cysts, fall outside the scope of the Bosniak system and require careful clinical correlation. *Conclusions*: By integrating CEUS into the diagnostic pathway, sonologists can achieve accurate lesion characterisation, optimise patient management, and minimise unnecessary invasive procedures, reinforcing CEUS as an essential tool in the evaluation and follow-up of complex renal cystic masses.

## 1. Introduction

Cysts are the most common kidney lesions identified in patients undergoing abdominal imaging, with ultrasound (US) typically serving as the initial diagnostic tool.

Simple cysts are the most frequent incidentally discovered lesions, with a prevalence of approximately 60–70%, while the prevalence of complex cysts is approximately 8–15% [1]. After contrast-enhanced computed tomography (CE-CT) and/or MRI, 5–10% of renal lesions remain indeterminate and require further characterisation [2]. While simple renal cysts exhibit characteristic features on greyscale US, evaluating renal lesions that do not meet the imaging criteria for simple cysts can be challenging. The first step in their assessment is to determine whether a renal lesion is solid or cystic and, if cystic, to evaluate its potential malignancy.

At US, malignancy may go undetected within non-tumoural echogenic content, and conversely, such echogenic material may mimic malignancy. Colour Doppler US identifies vascular lesions resembling cysts, such as aneurysms, and can assess the vascularity of septations and solid components in complex cystic lesions, but it often fails to detect slow blood flow in small vessels. Microvascular flow imaging techniques have been introduced, providing enhanced sensitivity for the evaluation of vascular flow and improving confidence in confirming the presence of vascularity [3,4,5], but assessment of tissue perfusion remains undetected. Therefore, lesions that are not unequivocally characterised as benign with B-mode US modes require further assessment after contrast agent administration, as the key feature that separates malignant from benign cystic lesions is determination of enhancing components, rather than morphological appearance [6,7].

## 2. Ultrasound Contrast Agents and Examination Technique

Contrast-enhanced ultrasound (CEUS) is the reference standard for assessing lesion vascularity [8,9]. It exploits the non-linear signals generated by the asymmetric oscillation of microbubbles and the marked acoustic impedance mismatch between microbubbles and blood, providing high sensitivity for the real-time detection of enhancement [10]. Renal masses that cannot be unequivocally characterised as benign on B-mode US require further contrast-enhanced evaluation [6,11,12,13]. CEUS has shown high diagnostic accuracy for the characterisation of cystic renal lesions, while avoiding ionising radiation [14,15,16,17,18,19,20,21].

Moreover, CEUS is not nephrotoxic, as microbubbles are not excreted by the kidneys, making it particularly suitable for patients with impaired renal function [22,23,24]. Growing evidence suggests that CEUS may be preferable to CE-CT and MRI [24,25,26,27,28,29,30,31].

**Figure 1:**
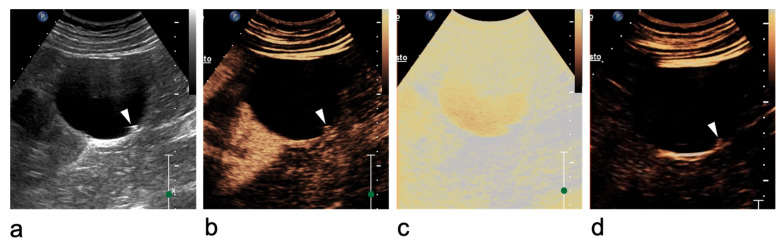
Ultrasound artefact mimicking an intracystic vegetation within a renal cyst at the lower pole of the right kidney. (**a**) Greyscale US shows focal thickening of the cystic wall (arrowhead). (**b**) On CEUS, enhancement of the focal thickening (arrowhead) is equivocal. (**c**) A high-mechanical index (MI) burst is applied to destroy microbubbles and determine whether the focal wall abnormality is vascularised. (**d**) After microbubble disruption, no change is observed (arrowhead), confirming the absence of vascularity. The wall thickening is therefore consistent with amorphous/intraluminal material rather than an enhancing vegetation (Bosniak category II cyst).

Careful technique is essential for renal lesion characterisation. Morphological and vascular features should first be assessed using B-mode US and Doppler imaging. After activation of the contrast-specific software, the mechanical index should be kept low, typically between 0.05 and 0.2, depending on the equipment. Microbubbles (SonoVue^®^, Bracco Imaging, Milan, Italy) are administered through a 20-gauge cannula as a bolus of 1.2 mL or less, followed by a 10 mL saline flush. During contrast circulation, the transducer should be moved to assess the entire lesion, and real-time video clips should be recorded and stored [20,32]. Excessive contrast injection should be avoided, as it may cause signal saturation and blooming artefacts, potentially leading to overscoring [33,34]. In equivocal cases, high-MI bursts can help confirm or exclude true vascularisation (Figure 1) [34].

## 3. Role of CEUS in Cystic Renal Lesions

**Figure 2:**
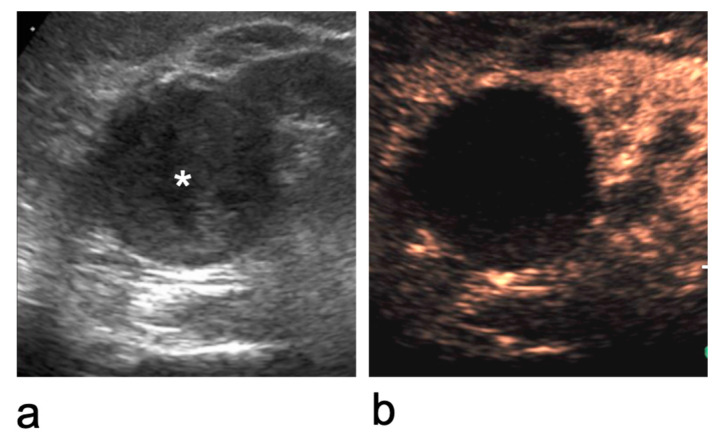
Characterisation of an indeterminate renal lesion on CEUS. (**a**) At the upper pole of the right kidney, an inhomogeneous iso- to hypoechoic lesion with regular margins is identified (asterisk). (**b**) After microbubble contrast injection, it is shown to be a benign cystic lesion with a thin, regular wall and no internal enhancing components (Bosniak category II cyst).

CEUS plays an established role in characterising renal lesions as cystic or solid across a range of clinical scenarios (Figure 2) including indeterminate lesions seen on B-mode US and hyperdense or equivocal findings on unenhanced or contrast-enhanced CT, where CEUS has shown high accuracy in differentiating solid from cystic masses and in reclassifying incidental renal lesions [35]. Furthermore, CEUS is valuable in the assessment of renal trauma, demonstrating parenchymal injuries, lacerations and, in some cases, active bleeding, complementing CT where appropriate, and it has demonstrated utility in inflammatory and infective renal pathologies such as abscesses [36].

## 4. Patients with Hyperdense Renal Lesions on CT

**Figure 3:**
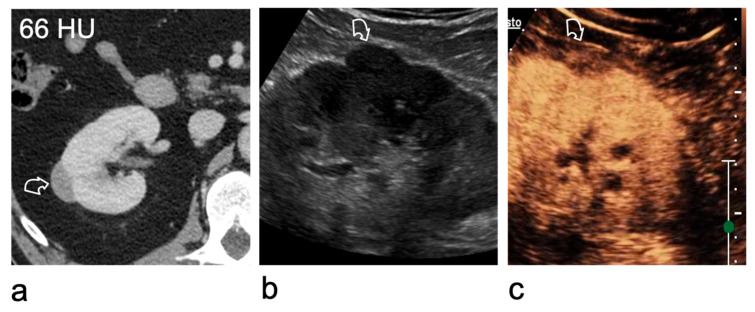
Characterisation of a right renal lesion indeterminate on contrast-enhanced CT. (**a**) Contrast-enhanced CT acquired only in the nephrographic phase shows a lesion (curved arrow) measuring 66 HU, which cannot be classified as solid or cystic because the unenhanced attenuation is unavailable. (**b**) On greyscale US, the lesion (curved arrow) remains equivocal as it is isoechoic to the renal parenchyma. (**c**) CEUS demonstrates that the lesion (curved arrow) is a solid, hypoenhancing tumour. A papillary renal cell carcinoma was found at surgery.

Lesions with densities of ≤20 HU or >70 HU are typically benign simple or haemorrhagic cysts [37]. However, lesions with densities between 20 and 70 HU may represent either benign cysts or tumours [38]. Hyperdense renal lesions may be identified incidentally on CT performed solely after contrast administration, and in the absence of pre-contrast imaging it can be challenging to differentiate benign cysts from solid lesions without further characterisation (Figure 3) [38,39]. In most cases, B-mode US is the next logical diagnostic step, as most high-attenuation benign renal cysts appear as simple cysts on US [40]. For lesions that remain indeterminate, contrast-enhanced imaging is crucial to evaluate vascularity [41]. CEUS is particularly well suited for this purpose, offering unmatched sensitivity in detecting blood flow.

## 5. Renal Lesions with Equivocal Enhancement on CE-CT

**Figure 4:**
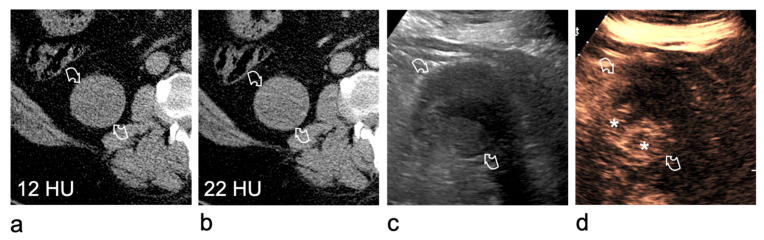
Utility of CEUS for characterising a right renal lesion (curved arrows) with equivocal enhancement on CT. (**a**) On the unenhanced scan, the right renal lesion measures 12 HU. (**b**) In the nephrographic phase after contrast administration, it shows equivocal enhancement (22 HU). (**c**) B-mode US remains non-diagnostic because the lesion is inhomogeneously echogenic. (**d**) CEUS unequivocally demonstrates an enhancing mural vegetation (asterisks), consistent with a Bosniak category IV lesion. Surgery revealed a low-grade clear cell renal cell carcinoma.

Occasionally, the solid or cystic nature of a renal lesion remains indeterminate despite a properly protocoled CT investigation, including unenhanced and contrast-enhanced nephrographic phases. While many tumours demonstrate unequivocal enhancement, some lesions enhance only minimally. In addition, simple cysts may show pseudoenhancement, namely an artefactual increase in attenuation of ≥10 HU. Renal masses demonstrating a 10–20 HU rise in attenuation may therefore represent either hypovascular tumours or cysts affected by pseudoenhancement. In this setting, CEUS is highly effective for the characterisation of lesions with equivocal enhancement on contrast-enhanced CT, demonstrating intralesional enhancement in virtually all solid lesions [24,41,42,43]. Consequently, CEUS reliably differentiates solid hypovascular tumours from cysts with pseudoenhancement, with high sensitivity and specificity (Figure 4).

## 6. The Bosniak Classification

The Bosniak classification system for cystic renal lesions was originally introduced in 1986 for use with contrast-enhanced CT and has subsequently undergone several revisions [25,44,45]. It is now widely and successfully applied to the categorisation of cystic renal lesions across other imaging modalities, including MRI and CEUS [46,47,48]. Although its application to CEUS dates back more than 20 years [49], several publications have appeared in recent years with the aim of better defining and validating the technique, standardising the procedure, and reducing inter- and intraobserver variability [2,8,25,50,51,52,53,54] (Table 1).

In summary, Bosniak categories I and II correspond to simple or minimally complicated benign cysts that do not require further evaluation. Category IIF is assigned to lesions that are presumed benign but warrant imaging surveillance because of indeterminate features, with follow-up aimed at confirming stability over time. A category III designation reflects diagnostic uncertainty, indicating that the lesion cannot be confidently classified as benign or malignant. Category IV denotes lesions considered highly suspicious for malignancy. Despite efforts to develop alternative scoring systems to address its limitations, the Bosniak classification remains the most widely accepted method for estimating the likelihood of malignancy in cystic renal lesions. Patient management depends on various factors, including imaging findings, clinical conditions, the patient’s age, and available treatment options. Characterising cystic renal lesions poses significant challenges, as the imaging boundary between benign and malignant entities is often poorly defined. This inherent ambiguity frequently leads to variability in interpretation among readers.

The Bosniak criteria are largely qualitative rather than quantitative, and the associated terminology is complex, which may hinder both learning and teaching. As a result, a high degree of interobserver variability persists, particularly in differentiating between Bosniak categories II, IIF and III, and the system remains strongly dependent on the reader’s level of experience [55,56,57]. Importantly, these limitations are largely independent of the imaging modality used to apply the Bosniak classification, whether CT, MRI or CEUS [15,16,58]. In response to these challenges, imaging parameters and definitions for category assignment have recently been standardised and validated, contributing to improved consistency and diagnostic accuracy [46,47,50,51,53].

## 7. Malignancy Rates for the Different Bosniak Classes

Determining the true prevalence of renal cell carcinoma across Bosniak categories is challenging, owing to uncertainty as to whether appropriate imaging techniques were used, whether the Bosniak classification was correctly applied, and the effects of selection and verification bias in surgical series [59]. An estimate of the malignancy risk has been discussed in detail by Silverman et al. [47]. Bosniak I cysts are considered benign, whereas Bosniak II cysts carry an extremely low risk of malignancy, generally regarded as close to 0 and below 1%, respectively. Bosniak IIF lesions show a low but non-negligible risk of malignancy, with reported rates varying widely from 0% to 38%. This broad range reflects important methodological limitations, since many Bosniak IIF lesions are managed conservatively and therefore lack histological confirmation, while surgical series are affected by strong selection and verification bias. Indeed, most Bosniak IIF lesions remain stable over time and do not progress, whereas lesions that are upgraded during follow-up to Bosniak III or IV carry a substantially higher likelihood of malignancy.

Bosniak III lesions remain the most problematic category, with approximately half of resected lesions proving malignant. Bosniak IV lesions carry the highest risk, with malignancy rates of around 90% in most series. These estimates should nevertheless be interpreted with caution, as reported rates are influenced by study design, imaging modality, reader experience, referral patterns, and, above all, by verification bias. Accordingly, Bosniak class should be regarded as an estimate of malignancy risk rather than as an absolute predictor of pathological outcome.

## 8. The Bosniak Classification in CEUS

In this narrative review, the Bosniak classification is referenced using the established CT/MRI framework released by Silverman et al. in 2019 [47]. For CEUS, we refer to the CEUS-adapted Bosniak criteria proposed by Cantisani et al. in 2020 [46]. These updated criteria introduce more explicit thresholds and definitions, including the number of septa, wall/septal thickness, and a standardised definition of mural nodules, to harmonise lesion assessment, address the well-recognised problem of interobserver variability in Bosniak classification, and thereby improve the consistency of category assignment. Categorisation on CEUS is driven primarily by the presence and morphology of enhancement of the wall, septa, mural nodules.

**Figure 5:**
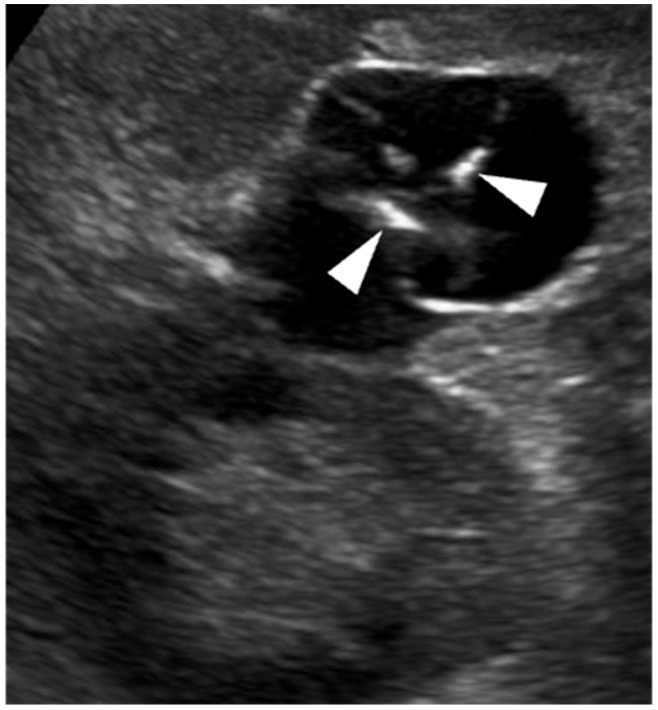
Bosniak category II left renal cyst that can be characterised on B-mode US. The cyst shows a few thin, partially calcified septa (arrowheads). Further characterisation with CEUS is not required.

Not all renal cysts require contrast administration for characterisation according to the Bosniak criteria. Category I cysts, as well as minimally complicated category II cysts with thin wall and septa without irregularities can be fully characterised using B-mode US alone (Figure 5) [46,60].

**Figure 6:**
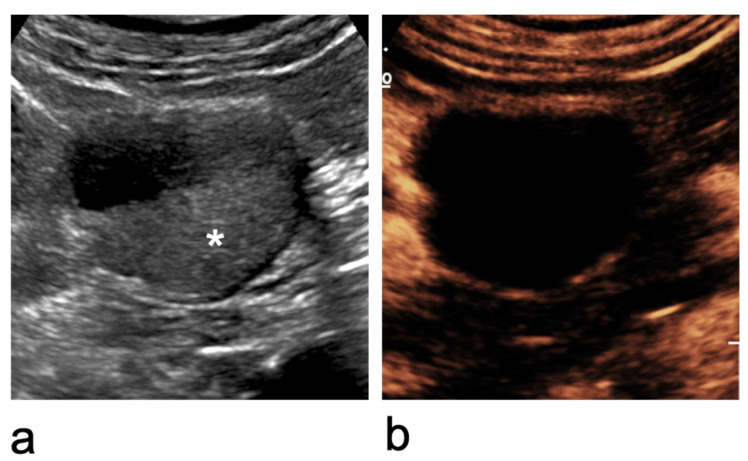
Bosniak category II cyst at the lower pole of the right kidney that cannot be characterised on B-mode US. (**a**) B-mode US shows a lesion with inhomogeneous internal echoes (asterisk). (**b**) CEUS demonstrates a benign cyst with a thin, smooth wall and no enhancing septa, wall irregularities, or mural nodules and vegetations.

**Figure 7:**
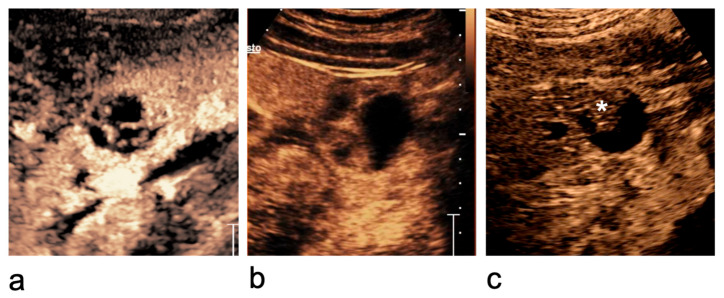
Bosniak category IIF–IV cystic lesions. (**a**) Bosniak category IIF cyst: completely intrarenal cyst with multiple thin or minimally thickened septa. (**b**) Bosniak category III cyst: cystic lesion with multiple thick, enhancing septa. (**c**) Bosniak category IV cystic lesion: presence of an enhancing vegetation (asterisk).

However, CEUS is recommended for the evaluation of cystic lesions that are well visualised on ultrasound but cannot be confidently characterised on B-mode US (Figure 6 and Figure 7) [46].

CEUS outperforms contrast-enhanced CT in detection of lesion vascularity, enabling the identification of a greater number of septa, improved depiction of wall and septal thickening, and more accurate assessment of septal enhancement and enhancement of solid components within lesions [16]. Owing to its very high sensitivity, CEUS is capable of depicting even minute capillaries supplying hairline septa, with superior temporal and spatial resolution compared with other imaging modalities. In light of these characteristics, there is a potential risk of overstaging benign cysts if these features are not appropriately interpreted within the Bosniak framework [33].

Although CEUS may depict septa and subtle enhancing components more clearly than CE-CT, higher sensitivity does not automatically translate into higher overall diagnostic accuracy. When applying a Bosniak-based approach with a more sensitive modality, the interpretation criteria must be adapted to the technique to avoid systematic upgrading (overscoring) driven by detection of minimal enhancement that may be below the detection threshold of CE-CT. For this reason, CEUS should not be regarded as a simple ‘CT-equivalent’, but rather as a modality requiring CEUS-adapted Bosniak criteria and careful integration with lesion morphology and clinical context [46]. In lesions that are well visualised on B-mode US, CEUS provides excellent lesion characterisation and can be considered at least comparable to MRI, while acknowledging that robust conclusions on comparative accuracy depend on reference standards (pathology and/or clinical outcome) and remain limited in parts of the literature.

**Figure 8:**
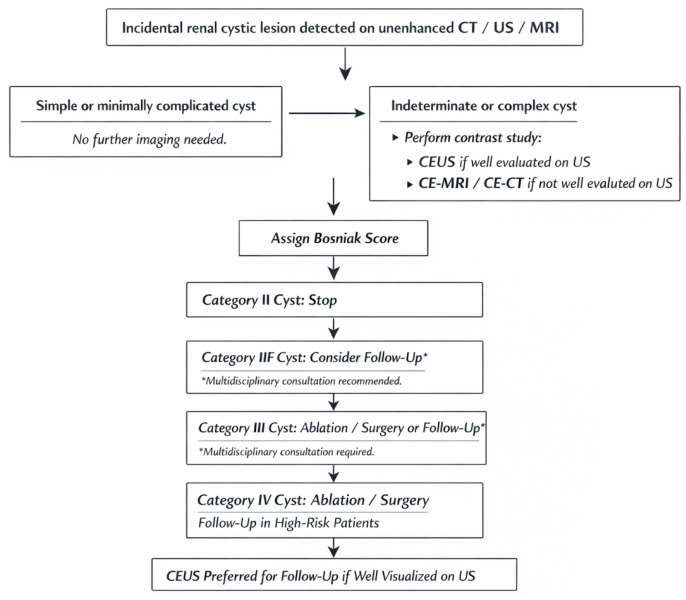
Simplified flowchart on the management of cystic renal lesions.

To address these limitations, CEUS imaging criteria for the assessment of cystic renal masses have recently been standardised, with the aim of improving interobserver agreement, reducing subjectivity, and minimising overscoring [44]. The criteria used to assign cystic renal lesions to Bosniak categories II–IV on CEUS are summarised in Table 2 [46]. The diagnostic pathway for incidental renal cystic lesion, including the role of CEUS and cross-sectional imaging, is summarised in Figure 8.

## 9. CEUS in Atypical Renal Cystic Masses in Children

Although the evidence base is smaller than in adults, largely because published paediatric series remain limited, the available data suggest that the safety profile of ultrasound contrast agents in children is broadly comparable to that observed in adults, with a very low rate of adverse reactions, while offering the important advantage of reducing exposure to ionising radiation [61]. The recommendations of the European Society of Paediatric Radiology Abdominal Imaging Task Force likewise emphasise that CEUS is a radiation-free, safe, portable and repeatable technique, with no nephrotoxicity, and is therefore particularly well suited to children [62]. Renal cysts are less common in children than in adults, with a reported prevalence of 0.22–2%, and the vast majority are simple; in the series by Karmazyn et al., complex cysts accounted for only 3.3–3.8% of cases [63]. With specific regard to the kidney, the imaging criteria used for the characterisation of cystic lesions are essentially analogous to those applied in adults according to the Bosniak classification [62].

CEUS should therefore be regarded as a particularly valuable tool in children for the characterisation and follow-up of cystic renal lesions, as it combines real-time assessment of vascularity with the major advantages of being radiation-free and non-nephrotoxic [62].

## 10. Reporting

The CEUS report should outline the practical circumstances of the examination, including technical adequacy and any factors that may have influenced image interpretation. Any circumstance that could limit diagnostic certainty should be clearly acknowledged, such as suboptimal acoustic windows related to patient morphology, deep lesion location, extensive calcifications obscuring intralesional assessment, or artefacts interfering with contrast evaluation. In some cases, complete visualisation of large cystic lesions may be precluded by bowel gas or adjacent anatomical structures. In fact, in a minority of cases, B-mode US may be insufficiently diagnostic to provide adequate lesion visualisation. In such circumstances, CEUS should not be attempted, and patients should proceed directly to contrast-enhanced CT or MRI, as their diagnostic yield is not affected by these ultrasound-specific limitations [19,34,36,46].

When CEUS is performed, a cine clip of the contrast-enhanced examination should be recorded and stored to allow subsequent review of enhancement dynamics. B-mode US findings should be described separately, documenting morphological characteristics such as internal septations, calcified walls or septa, and the nature of the cyst contents. CEUS assessment should then focus on contrast behaviour, reporting the presence or absence of enhancement within the cyst wall, septa or any intralesional solid elements, as well as the distribution and degree of wall or septal irregularity [46].

## 11. Lesions to Which the Bosniak Classification System Is Not Applicable

In general, percutaneous biopsy of cystic renal lesions is not recommended because of the relatively high rate of non-diagnostic samples and concerns regarding potential cyst rupture and tumour seeding. An exception may be represented by biopsy of a clearly identifiable solid component within a Bosniak IV cystic lesion [64]. In selected cases, biopsy of cystic renal masses may also be considered to obtain a tissue diagnosis in patients who are poor surgical candidates or prior to image-guided ablative therapies, when histological confirmation of malignancy is required to guide management [65].

After percutaneous drainage and sclerotherapy, cyst morphology may be substantially altered by reactive or inflammatory changes, potentially mimicking increased complexity. In this setting, the Bosniak classification, developed for native, untreated cystic renal lesions, should not be applied, as post-procedural appearances do not reflect true malignant risk [66].

**Figure 9:**
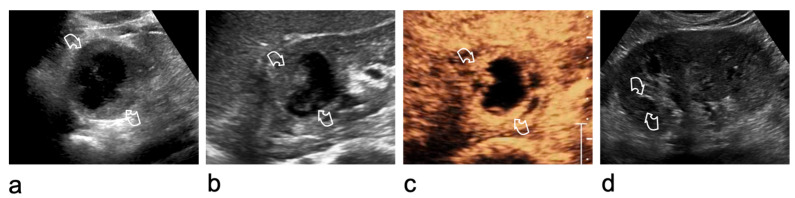
Inflammatory renal lesion in which the Bosniak classification is not applicable. A 54-year-old woman presenting with fever, left flank pain, and leukocytosis. (**a**) At the upper pole of the left kidney, a 4.4 × 3.2 cm complex cystic lesion is seen (curved arrows), with a thickened, irregular wall and echogenic content. Given the clinical context, an infected cyst or renal abscess was suspected. (**b**,**c**) Greyscale US and CEUS performed after 7 days of antibiotic therapy show a reduction in lesion size (2.8 × 2.2 cm) but persistent complexity (curved arrows). (**d**) At 6-month follow-up, a 1 cm simple cyst is seen at the same site (curved arrows), consistent with resolution of inflammation.

Other scenarios in which the Bosniak classification system is inapplicable include traumatic cystic rupture, infected cysts and renal abscesses (Figure 9). These entities, although benign, often fulfil imaging criteria that would otherwise suggest a Bosniak III lesion, such as thickened enhancing wall with irregularities [67,68]. In such cases, a detailed clinical history and correlation with laboratory findings are essential to differentiate these conditions from cystic neoplasms, as imaging features alone may be misleading.

Finally, it is important to emphasise that the Bosniak classification system is specifically intended for cystic renal lesions, defined as masses in which enhancing tissue accounts for less than approximately 25% of the overall volume [47]. It should not be used for solid lesions with extensive necrotic changes mimicking cystic components. These solid lesions differ significantly from cystic tumours in their natural history, as they are typically aggressive and associated with a poor prognosis [47].

## 12. Follow-Up

The Bosniak classification is currently the best available tool for risk stratification of cystic renal lesions, as it provides an imaging-based estimate of malignancy risk. Earlier versions of the classification tended to imply a direct link between Bosniak category and management, such as discharge, imaging surveillance, or surgery. However, Bosniak classification should not be interpreted as a rigid therapeutic algorithm. Its primary role is not to dictate treatment, but to provide the urologist and the multidisciplinary team with reliable information on the likelihood of malignancy. Clinical management should therefore be individualised and based on an overall patient assessment, including age, comorbidities, surgical fitness, life expectancy, patient preferences, and local expertise. Accordingly, although Bosniak I and II cysts usually require neither treatment nor follow-up, Bosniak IIF lesions are usually followed-up, but may occasionally be considered for intervention, whereas observation may be appropriate for some Bosniak III lesions, particularly in elderly, frail, or comorbid patients, with acceptable oncological outcomes reported in some series [59,69,70]. Even Bosniak IV lesions may, in exceptional circumstances, be managed non-operatively when surgery is not feasible or would not be in the patient’s best interest.

Within this framework, CEUS is particularly well suited to the follow-up of non-surgical cystic renal lesions because it is repeatable, cost-effective, and free of ionising radiation. The optimal follow-up schedule remains controversial and represents an unmet clinical need [8,9,14]. In clinical practice, follow-up is often performed at 6 months, 12 months, and then annually for up to 5 years, depending on lesion complexity and patient-related factors [71,72]. Shorter surveillance strategies of 2–3 years have also been proposed [28,30,73,74]. During follow-up, lesion growth alone is not considered a criterion for progression; more relevant is an increase in imaging complexity with upgrading of the Bosniak category. Reported progression rates for Bosniak IIF lesions are generally low, although variable across studies, and progression appears more likely to occur within the first 2 years after detection [47,72,75,76,77].

## 13. Summary Considerations

CEUS offers a non-invasive, highly sensitive method for characterising cystic renal lesions, especially in cases where B-mode US or CE-CT results are inconclusive. The Bosniak classification system should not be applied indiscriminately to lesions where significant morphological changes have occurred due to interventions, traumas or infections. Standardised imaging parameters can improve interobserver agreement, reduce subjectivity, and enhance diagnostic accuracy. By incorporating these best practices, sonologists can optimise the use of CEUS in the diagnosis and management of cystic renal lesions, ensuring accurate assessments while reducing unnecessary interventions.

Future perspectives include further prospective multicentre studies, as well as systematic reviews and meta-analyses, to strengthen the evidence base on safety, reproducibility and clinical utility in both adults and children. This, in turn, may support broader regulatory approval and reimbursement across countries [78]. Continued technological advances, particularly in super-resolution ultrasound imaging and artificial intelligence-assisted quantitative analysis, are expected to improve spatial and contrast resolution, standardisation, lesion characterisation and diagnostic confidence [79].

## Figures and Tables

**Table 1:** Relevant publications of the last 5 years.

First Author	Year	Title	Reference
Schwarze et al.	2020	Contrast-Enhanced Ultrasound (CEUS) for the Evaluation of Bosniak III Complex Renal Cystic Lesions- A 10-Year Specialized European Single-Center Experience with Histopathological Validation	[50]
Münch et al.	2020	Intra- and Interobserver Study Investigating the Adapted EFSUMB Bosniak Cyst Categorization Proposed for Contrast-Enhanced Ultrasound (CEUS) in 2020	[51]
Cantisani et al.	2021	EFSUMB 2020 Proposal for a Contrast-Enhanced Ultrasound-Adapted Bosniak Cyst Categorization—Position Statement	[46]
Como et al.	2021	Role of contrast-enhanced ultrasound in assessing indeterminate renal lesions and Bosniak ≥2F complex renal cysts found incidentally on CT or MRI	[2]
Herms et al.	2023	Ultrasound-based ‘CEUS-Bosniak’ classification for cystic renal lesions: an 8-year clinical experience	[52]
Jin et al.	2023	Contrast-enhanced US Bosniak Classification: intra- and inter-rater agreement, confounding features, and diagnostic performance	[53]
Möller et al.	2023	CEUS Bosniak Classification-Time for Differentiation and Change in Renal Cyst Surveillance	[25]
Járay et al.	2024	The Predictive Power of Bosniak 3 and 4 Cystic Renal Lesion Categorization Using Contrast-Enhanced Ultrasound	[54]
Járay et al.	2025	The value of contrast-enhanced ultrasound in the follow-up of Bosniak IIF cystic renal lesions	[8]

**Table 2:** Bosniak renal cyst classification on B-mode and CEUS (modified from Reference [46]).

B-Mode Appearance	CEUS	CEUS Appearance	Bosniak Category
Simple cyst	Not required	Wall is smooth and usually non-enhancing. When enhancement is present, few microbubbles are seen coursing within thin vessels in the wall	I
Cyst otherwise meeting criteria for a simple cyst but with 1–3 thin septa (<2 mm) without irregularities. Wall and/or septal calcifications may be present, provided they do not impede assessment of cyst contents	Not required	Wall and septa are smooth and usually non-enhancing. When enhancement is present, few microbubbles are seen coursing within the mural/septal vessels	II
Cysts with internal debris, echogenic content, or mixed internal echoes	Required	Wall and any septa are smooth and usually non-enhancing. When enhancement is present, few microbubbles are seen coursing within the mural/septal vessels	
Cyst with multiple septa and/or internal debris, echogenic content, or mixed appearance. Calcifications may be present and may slightly limit evaluation of the wall, septa and contents	Required	Septa: Multiple enhancing septa, thin or minimally thickened (2–3 mm), smooth or with only minor irregularity Wall: Minimally thickened enhancing wall (2–3 mm), predominantly smooth or with only minor irregularity	IIF
Completely intrarenal cyst otherwise consistent with category II features	Required	Thin or minimally thickened (2–3 mm) enhancing septa may be present. In completely intraparenchymal cysts, differentiating wall enhancement from enhancement of the adjacent renal parenchyma is unreliable, as the cyst wall cannot be clearly separated from the surrounding enhancing tissue	
Cyst with multiple septa and/or internal debris, echogenic content, or mixed appearance	Required	Enhancing thick wall and/or septa, smooth (≥4 mm) or irregular (>3 mm). No enhancing nodules	III
Cyst with multiple septa and/or internal debris, echogenic content, or mixed appearance	Required	Findings as in category III plus enhancing nodules with obtuse margins (≥4 mm) or with acute margins of any size	IV

## Data Availability

No new data were created or analysed in this study. Date sharing is not applicable to this article.

## Abbreviations

The following abbreviations are used in this manuscript:

CEUS	Contrast-enhanced ultrasound
US	Ultrasound
CE-CT	Contrast-enhanced computed tomography
MI	Mechanical index
